# Réduction de la luxation de la tête radiale dans le cadre de la maladie exostosante: à propos d'un cas

**DOI:** 10.11604/pamj.2015.20.95.5849

**Published:** 2015-02-04

**Authors:** Malek Meherzi, Mourad Jenzri, Aymen Zaier, Moez Kaaniche, Zied Jlailia, Khaled Kamoun, Omar Zouari

**Affiliations:** 1 Faculté de Médecine de Tunis, Université de Tunis El Manar, Service d'Orthopédie Infantile-Institut MT Kassab, Tunisie

**Keywords:** Exostoses, avant-bras, tête radiale, allongement des os, Exostoses, forearm, radial heads, bone lengthening

## Abstract

La luxation de la tête radiale dans le cadre de la maladie exostosante constitue une complication de déséquilibre de croissance des deux os de l'avant bras secondaire à une exostose distale de l'ulna. Le traitement est difficile et controversé par les auteurs. Nous présentons une technique originale pour le traitement de cette entité. Après la correction de l'index radio-cubital inférieur par un allongement progressif de l'ulna, une fixation radio-cubitale inférieure par les fiches distales de l'Orthofix^®^ et un deuxième allongement de l'ulna permet l'abaissement de la tête radiale et sa réduction. La réduction progressive par allongement de l'ulna par la méthode de callotasis constitue une technique séduisante et réalise l'inverse du ce qui passé lors de ralentissement de croissance de l'ulna sous l'effet de l'exostose ulnaire.

## Introduction

La maladie des exostoses multiples a été décrite en premier par Bayer en 1814, elle représente la tumeur bénigne osseuse la plus fréquente chez l'enfant. Sa transmission est autosomale dominante avec une pénétrance variable [1]. La localisation la plus commune des ostéochondromes est la métaphyse des os longs et atteint l'avant bras dans 30 à 60% des cas [2]. L'extrémité distale de l'ulna est électivement la plus touchée, provoque un raccourcissement ulnaire, une incurvation radiale et une dislocation de la radio-ulnaire inférieure. L’évolution sans traitement se fait parfois vers une luxation de la tête radiale augmentant ainsi la gêne fonctionnelle et le préjudice esthétique. Les principes chirurgicaux restent un sujet de controverse et les procédés de réduction de la tête radiale sont peu décris dans la littérature avec des résultats aléatoires. Nous présentons une technique originale de réduction de la tête radiale chez une patiente porteuse d'une déformation de l'avant bras type IIb (Masada) [3].

## Patient et observation

**Figure 1:**
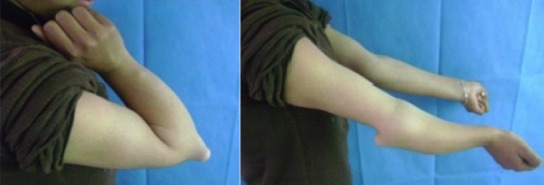
Déformation de l'avant bras et le déficit de mobilité en préopératoire

**Figure 2:**
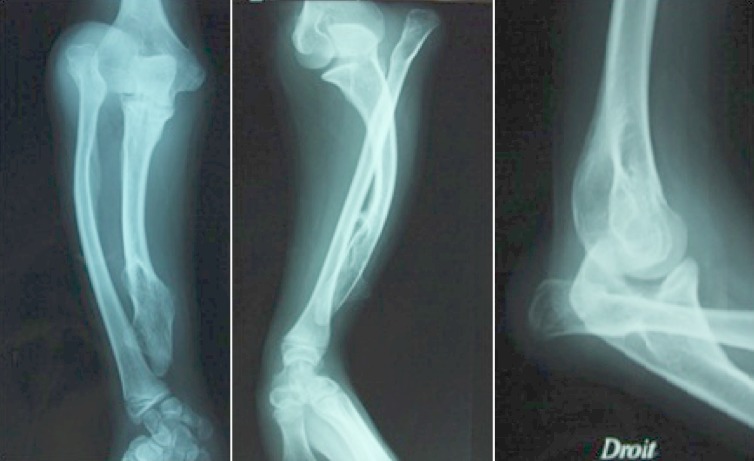
Les radiographies de face et de profil montre l'exostose ulnaire inférieure et la luxation de la tête radiale

Il s'agissait d'une élève droitière âgée de 15 ans, suivie pour des exostoses multiples et présentant un flessum de 20° du coude droit, une déformation de l'avant, une main botte cubitale et une tuméfaction solide postérieure au niveau du coude. La supination-pronation était à 60°/70° (Figure 1). La radiographie a montré une exostose de l'extrémité distale de l'ulna qui est raccourci, un radius incurvé et une luxation de la tête radiale (Masada IIb). Le raccourcissement ulnaire était de 9mm, l'inclinaison radiale était de 42° et la translation ulnaire du carpe était de 60% [4] (Figure 2).

**Figure 3:**
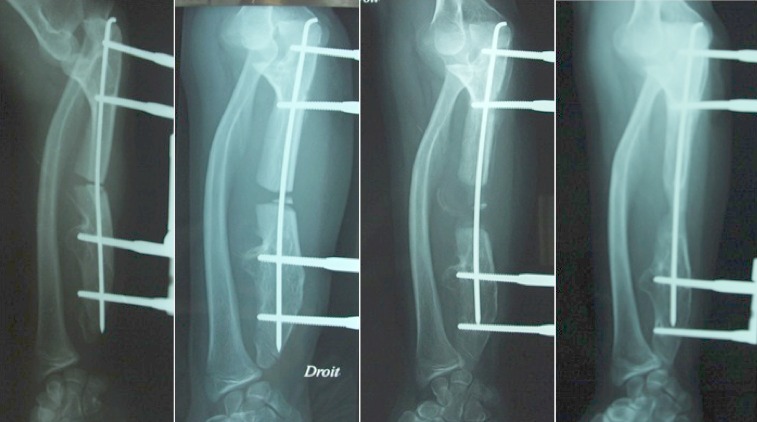
Progression de l'allongement de l'ulna et persistance de la luxation de tête radiale

**Figure 4:**
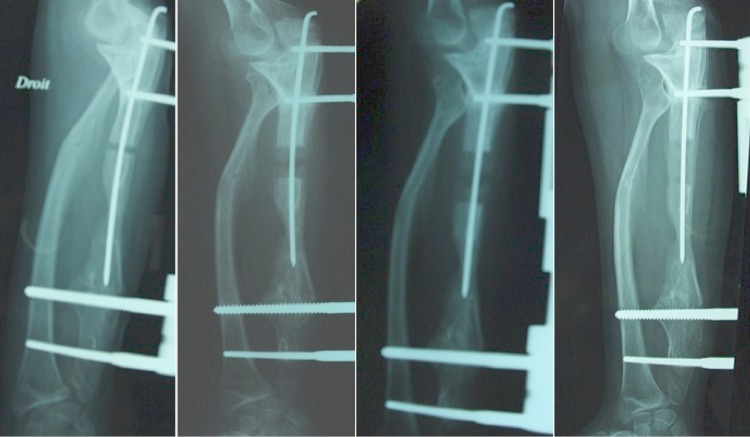
Fixation de la radio-ulnaire inférieure par les broches distales et réduction progressive de la tête radiale

**Figure 5:**
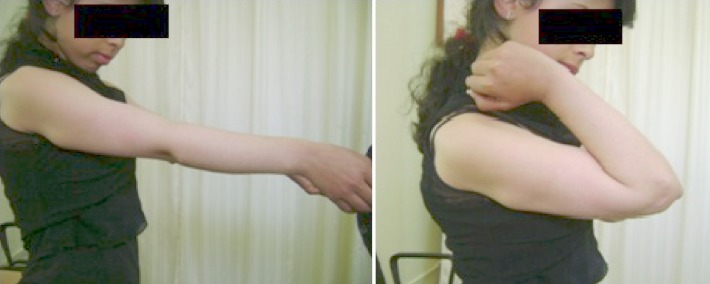
Correction de la déformation de l'avant-bras, amélioration de la mobilité et disparition de la tuméfaction du coude en post-opératoire

Dans un but de correction de la déformation du poignet et le rétablissement de l'index radio-cubital inférieur, on a procédé d'abord à l'allongement de l'ulna par un Orthofix^®^. Une ostéotomie transversale a été pratiquée en medio-diaphysaire. L'allongement a été débuté après 10 jours à raison de 1 mm par jour en 4 fois. Après un allongement de 12mm la correction de l'index radio-cubital inférieur était satisfaisante (Figure 3) avec persistance de la luxation de la tête radiale. Dans un deuxième temps on a fixé les deux os de l'avant bras par le groupe de fiches distales de l'Orthofix^®^, pour réaliser une deuxième ostéotomie ulnaire et l'allongement progressif de l'ulna permettant l'abaissement de la tête radiale. Après un allongement de 18mm, la tête radiale était au même niveau que l'apophyse coronoîde sur les clichés de profil (Figure 4). L'Orthofix^®^ a été enlevé après consolidation du régénérat et une rééducation de 6 semaines a été prescrite. A 14 mois post opératoire, la tuméfaction du coude a disparu, la patiente présente une extension complète du coude et une supination-pronation à 30°/80° (Figure 5). L'inclinaison radiale mesurée sur les radiographies de contrôle est de 35° et la translation ulnaire du carpe est de 50%.

## Discussion

Les recommandations des auteurs pour les indications et les délais optimaux de la chirurgie dans le traitement des déformations de l'avant-bras dans le cadre de la maladie exostosante sont très controversées. Fogel et al [4] et Peterson et al [5] insistent sur la nécessité d'un traitement précoce et agressif afin de prévenir l'exagération des déformations et l'handicap fonctionnel secondaire. D'après l’étude d'une série d'adulte non traités, Noonan et al [6] concluent que les patients gardent une activité quotidienne satisfaisante et justifient le traitement chirurgical par un bénéfice esthétique seulement. Les travaux publiés portant sur les modalités de réduction de la tête radiale dans les déformations Masada IIa et IIb sont rares. Dans la série étudiée par la SOFCOT en 1987, un peu plus de la moitié des cas ont eu une réduction de la tête radiale après l'allongement de l'ulna [7]. Certains auteurs préconisent une résection de la tête radiale à l’âge adulte sans influence sur la mobilité du coude et du poignet [8]. Trois cas dans la série d'Akita et al [9] ont bénéficié d'une réduction à ciel ouvert de la tête radiale avec une reconstruction du ligament annulaire. L’évaluation de la mobilité chez ces patients à long terme a montré une restriction de la pronation. Parmi les six patients traités par Demir et al [10], trois étaient classés Masada IIb et sont les seuls traités par une technique comparable à la notre dans la littérature. Le montage utilisé chez deux patients était un Ilizarov et un montage combiné par deux fixateurs monoplans solidarisés. L'avantage de la fixation monolatérale par Orthofix^®^utilisé chez notre patiente est l'absence de l'encombrement du membre qui pourrait compromettre l'adhésion des enfants à ce traitement.

## Conclusion

La réduction de la tête radiale secondaire à une exostose distale de l'ulna est très peu décrite dans la littérature. L'allongement isolé de l'ulna peu s'avérer insuffisant, dans ce cas nous préconisons la fixation de la radio-ulnaire inférieure par un seul montage d'Orthofix^®^.

## References

[1] Unni KK (1996). Dahlin's Bone Tumors: General Aspects and Data on 11,087 Cases. Lippincott-Raven.

[2] Jaffe HL (1943). Hereditary multiple exostosis. Arch Pathol..

[3] Masada K, Tsuyuguchi Y, Kawai H, Kawabata H, Noguchi K, Ono K (1989). Operations for forearm deformity caused by multiple osteochondromas. The Journal of bone and joint surgery British volume.

[4] Fogel GR, McElfresh EC, Peterson HA, Wicklund PT (1984). Management of deformities of the forearm in multiple hereditary osteochondromas. The Journal of bone and joint surgery American volume.

[5] Peterson HA (1994). Leg length discrepancy associated with vivid cutis marmorata. Journal of pediatric orthopedics.

[6] Noonan KJ, Levenda A, Snead J, Feinberg JR, Mih A (2002). Evaluation of the forearm in untreated adult subjects with multiple hereditary osteochondromatosis. The Journal of bone and joint surgery American volume.

[7] Caton J, Kohler R, Fournet-Fayard J, Berard J, Michel CR (1988). [Progressive lengthening of the ulna in children using Wagner's technic. Apropos of 14 cases]. Rev Chir Orthop Reparatrice Appar Mot..

[8] Wood VE, Sauser D, Mudge D (1985). The treatment of hereditary multiple exostosis of the upper extremity. The Journal of hand surgery.

[9] Akita S, Murase T, Yonenobu K, Shimada K, Masada K, Yoshikawa H (2007). Long-term results of surgery for forearm deformities in patients with multiple cartilaginous exostoses. The Journal of bone and joint surgery American volume.

[10] Demir B, Gursu S, Ozturk K, Yildirim T, Konya MN, Er T (2011). Single-stage treatment of complete dislocation of radial head and forearm deformity using distraction osteogenesis in paediatric patients having multiple cartilaginous exostosis. Archives of orthopaedic and trauma surgery.

